# Supplementary material for: Acupuncture for military veterans with posttraumatic stress disorder and related symptoms after combat exposure: Protocol for a scoping review of clinical studies
Source: PLoS One. 2023 Apr 21;18(4):e0273131. doi: 10.1371/journal.pone.0273131 (PMC10120924; doi:10.1371/journal.pone.0273131)
Supplement: S1 Table — (DOCX) [file pone.0273131.s002.docx]

**S1 Supplementary digital content. Search terms used in each database**

**Medline via PubMed**

|  | Searches | Results |
| --- | --- | --- |
| #1 | (“War Exposure”[MeSH] OR “Armed Conflicts” [MeSH] OR “Veterans”[MeSH] OR “Military Personnel“[MeSH] OR “Combat Disorders”[MeSH] OR War exposure[Title/abstract] OR Armed Conflicts[Title/abstract] OR Veterans[Title/abstract] OR Military Personnel[Title/abstract] OR war[Title/abstract] OR Combat[Title/abstract]) |  |
| #2 | ("Acupuncture Therapy"[MeSH] OR "Acupuncture, Ear"[MeSH] OR "Acupuncture Points"[MeSH] OR "Acupuncture"[MeSH] OR "Electroacupuncture"[MeSH] OR "Meridians"[MeSH] OR acupuncture[Title/abstract] OR electroacupuncture[Title/abstract] OR electro-acupuncture[Title/abstract] OR acupoint*[Title/abstract]) |  |
| #3 | #1 AND #2 |  |

**EMBASE via Elsevier**

|  | Searches | Results |
| --- | --- | --- |
| #1 | ‘war exposure’/exp OR ‘war exposure’:ab,ti OR ‘armed conflicts’:ab,ti OR ‘war’/exp OR ‘war’:ab,ti OR ‘Veteran’/exp OR ‘Veterans’:ab,ti OR ‘Military Personnel’/exp OR ‘Military Personnel’:ab,ti OR ‘Combat stress’/exp OR ‘Combat’:ab,ti |  |
| #2 | (‘acupuncture’/exp OR ‘acupuncture’:ab,ti OR ‘acupuncture therapy’:ab,ti OR ‘auricular acupuncture’/exp OR ‘auricular acupuncture’:ab,ti OR ‘ear acupuncture’:ab,ti OR ‘acupuncture point’/exp OR ‘acupuncture point’:ab,ti OR ‘electroacupuncture’/exp OR ‘electroacupuncture’:ab,ti OR ‘electro-acupuncture’:ab,ti OR ‘body meridian’/exp OR ‘body meridian’:ab,ti OR ‘acupoint’:ab,ti) |  |
| #3 | #1 AND #2 |  |

**CENTRAL**

|  | Searches | Results |
| --- | --- | --- |
| #1 | MeSH descriptor: [Armed Conflicts] explode all trees |  |
| #2 | MeSH descriptor: [Veterans] explode all trees |  |
| #3 | MeSH descriptor: [Military Personnel] explode all trees |  |
| #4 | MeSH descriptor: [Combat Disorders] explode all trees |  |
| #5 | MeSH descriptor: [War Exposure] explode all trees |  |
| #6 | (armed conflicts OR veterans OR military personnel OR combat OR war OR war exposure):ti,ab,kw |  |
| #7 | #1 OR #2 OR #3 OR #4 OR #5 OR #6 |  |
| #8 | MeSH descriptor: [Acupuncture] explode all trees |  |
| #9 | MeSH descriptor: [Acupuncture Therapy] explode all trees |  |
| #10 | MeSH descriptor: [Acupuncture, Ear] explode all trees |  |
| #11 | MeSH descriptor: [Electroacupuncture] explode all trees |  |
| #12 | (Acupuncture OR Pharmacopuncture OR Ear acupuncture OR Pharmacoacupuncture OR Electroacupuncture OR Acupotomy OR Acupotomies OR Ear acupuncture OR Auricular acupuncture):ti,ab,kw |  |
| #13 | #8 OR #9 OR #10 OR #11 OR #12 |  |
| #14 | #7 AND #13 in Trials |  |

**Web of Science**

|  | Searches | Results |
| --- | --- | --- |
| #1 | (TS=(war exposure) OR TS=(war) OR TS=(armed conflicts) OR TS=(veterans) OR TS=(military personnel) OR TS=(combat disorders) OR TS=(combat)) |  |
| #2 | (TS=(aupuncture therapy) OR TS=(acupuncture) OR TS=(acupuncture point) OR TS=(auricular acupuncture) OR TS=(ear acupuncture) OR TS=(electroacupuncture) OR TS=(electro-acupuncture) OR TS=(meridian) OR TS=(acupoint*)) |  |
| #3 | #1 AND #2 |  |

**Scopus**

|  | Searches | Results |
| --- | --- | --- |
| #1 | (TITLE-ABS-KEY (war exposure) OR TITLE-ABS-KEY (war) OR TITLE-ABS-KEY (armed conflicts) OR TITLE-ABS-KEY (veterans) OR TITLE-ABS-KEY(military personnel) OR TITLE-ABS-KEY (combat disorders) OR TITLE-ABS-KEY(combat)) |  |
| #2 | (TITLE-ABS-KEY (aupuncture therapy) OR TITLE-ABS-KEY (acupuncture) OR TITLE-ABS-KEY (acupuncture point) OR TITLE-ABS-KEY (auricular acupuncture) OR TITLE-ABS-KEY (ear acupuncture) OR TITLE-ABS-KEY (electroacupuncture) OR TITLE-ABS-KEY (electro-acupuncture) OR TITLE-ABS-KEY (meridian) OR TITLE-ABS-KEY (acupoint*)) |  |
| #3 | #1 AND #2 |  |

**AMED via EBSCO**

|  | Searches | Results |
| --- | --- | --- |
| #1 | (war exposure[TX] OR war[TX] OR armed conflicts[TX] OR veterans[TX] OR military personnel[TX] OR combat disorder[TX] OR combat[TX]) |  |
| #2 | (“Acupuncture Therapy”[SU] OR “Acupuncture, Ear”[SU] OR “Acupuncture Points”[SU] OR Acupuncture[SU] OR Electroacupuncture[SU] OR Meridians[SU] OR acupuncture[TX] OR electroacupuncture[TX] OR electro-acupuncture[TX] OR acupoint*[TX]) |  |
| #3 | #1 AND #2 |  |

**CINAHL via EBSCO**

|  | Searches | Results |
| --- | --- | --- |
| #1 | (MH “War+”) OR (MH “Veterans+”) OR (MH “Military personnel+”) OR (TX war exposure) OR (TX war) OR (TX armed conflicts) OR (TX veterans) OR (TX military personnel) OR (TX combat disorder) OR (TX combat) |  |
| #2 | (MH “Acupuncture+”) OR (MH “Acupuncture, Ear+”) OR (MH “Acupuncture Points+”) OR (MH “Electroacupuncture”) OR (MH “Meridians+”) OR (TX acupuncture) OR (TX electroacupuncture) OR (TX electro-acupuncture) OR (TX acupoint*) |  |
| #3 | #1 AND #2 |  |

**PsycARTICLES via ProQuest**

|  | Searches | Results |
| --- | --- | --- |
| #1 | MAINSUBJECT.EXACT.EXPLODE("Military Personnel") OR MAINSUBJECT.EXACT.EXPLODE("Military Veterans") OR MAINSUBJECT.EXACT.EXPLODE("Combat Experience") OR title(war exposure) OR abstract(war exposure) OR title(war) OR abstract(war) OR title(armed conflicts) OR abstract(armed conflicts) OR title(veterans) OR abstract(veterans) OR title(military personnel) OR abstract(military personnel) OR title(combat disorder) OR abstract(combat disorder) OR title(combat) OR abstract(combat) |  |
| #2 | MAINSUBJECT.EXACT.EXPLODE("Acupuncture") OR title(acupuncture) OR abstract(acupuncture) OR title(acupuncture therapy) OR abstract(acupuncture therapy ) OR title(ear acupuncture) OR abstract(ear acupuncture) OR title(acupuncture points) OR abstract(acupuncture points) OR title(acupoint*) OR abstract(acupoint*) OR title(electroacupuncture) OR abstract(electroacupuncture) OR title(electro-acupuncture) OR abstract(electro-acupuncture) OR title(meridians) OR abstract(meridians) |  |
| #3 | #1 AND #2 |  |

**OASIS**

|  | Searches | Results |
| --- | --- | --- |
| #1 | (전쟁 OR 전투 OR 무력충돌 OR 군대 OR 군인) AND 침 |  |

**KCI**

|  | Searches | Results |
| --- | --- | --- |
| #1 | (전쟁 OR 전투 OR 무력충돌 OR 군대 OR 군인) AND 침 |  |

**CNKI**

|  | Searches | Results |
| --- | --- | --- |
| #1 | (SU='战争'+’战斗’+’武装冲突’ +’军队’+’军人’) AND (SU='acupuncture'+'针'+'鍼') |  |

**Wanfang data**

|  | Searches | Results |
| --- | --- | --- |
| #1 | ((((主题=战争) OR 主题=战斗) OR 主题=武装冲突) OR 主题=军队) OR 主题=军人) AND (((主题=acupuncture) OR 主题=针) OR 主题=鍼) |  |

**VIP**

|  | Searches | Results |
| --- | --- | --- |
| #1 | M=(战争 OR 战斗 OR 武装冲突 OR 军队 OR 军人) AND M=(acupuncture OR 针 OR 鍼) |  |

**CiNii**

| CiNii | Searches | Results |
| --- | --- | --- |
| #1 | (戦争 OR 戦闘 OR 武力衝突 OR 軍隊 OR 軍人) AND (acupuncture OR 針 OR 鍼) |  |
